# Acute-on-Chronic Pancreatitis: Analysis of Clinical Profile and Outcome

**DOI:** 10.7759/cureus.14242

**Published:** 2021-04-01

**Authors:** Dibyajyoti Sharma, Bipadabhanjan Mallick, Jayanta Samanta, Vikas Gupta, Saroj K Sinha, Rakesh Kochhar

**Affiliations:** 1 Internal Medicine, Gastroenterology, Silchar Medical College and Hospital, Silchar, IND; 2 Gastroenterology, Kalinga Institute of Medical Sciences, Bhubaneswar, IND; 3 Gastroenterology, Postgraduate Institute of Medical Education and Research, Chandigarh, IND; 4 General Surgery, Postgraduate Institute of Medical Education and Research, Chandigarh, IND

**Keywords:** acute-on-chronic pancreatitis, clinical profile, outcome

## Abstract

Objective: Overall, a handful of studies are available on the outcomes of acute-on-chronic pancreatitis (ACP). We aimed to provide a more complete and updated picture of ACP.

Methods: We evaluated consecutive patients of acute exacerbation of chronic pancreatitis (CP) in a tertiary care center located in north India and studied their epidemiological profiles, etiological factors as well as outcomes.

Results: Forty-five patients of ACP with a mean age of 37±13 years were evaluated. The majority of the patients were male (75%) and alcohol was the most common detectable etiology while no etiology could be identified in 35% of patients after extensive laboratory investigations and imaging. Moderately severe pancreatitis was noted in 73% of patients and 49% of patients had necrotizing pancreatitis out of which the majority (33%) had both pancreatic as well as extra-pancreatic necrosis (EPN). Five patients (11%) were subjected to percutaneous catheter drainage. Persistent organ failure was noted in 9% of patients and two (4.5%) patients had died from organ failure.

Conclusion: To conclude, this study has demonstrated that ACP has a milder disease course and low morbidity and mortality. Early elimination of the etiological factor is essential for optimal outcome.

## Introduction

Acute and chronic pancreatitis are two spectrums of the same disease process and they have common etiologies like alcohol abuse, drugs, metabolic causes like hyperparathyroidism and hypertriglyceridemia. Studies have suggested that 10-35% of acute pancreatitis (AP) patients who survive the initial attack go on to develop recurrent attacks of pancreatitis (RAP) [1] and some of these patients (6-13%) may progress to chronic pancreatitis (CP) [2]. Lankisch et al. [2] had observed that progression to CP from AP only in alcoholics with an increasing trend co-related with the duration of disease (13% in 10 years and 16% in 20 years) and 38% of the patients developed CP within two years of surviving the second attack of pancreatitis. However, the Danish registry has also reported progression to CP after an initial attack of AP in patients with etiology other than alcohol. Progressive AP has higher mortality compared to the general population by four to six times [3,4]. The cause of death in CP was attributed to digestive diseases (CP itself and liver diseases), cancer and cardiovascular ailments, out of which pancreatic cancer was detected in 3.8% of patients [4,5]. The course of disease and severity of pancreatitis between acute-on-chronic pancreatitis (ACP) and the initial attack of AP was contrasting. Patients with underlying CP have a smoldering disease course with recurrent episodes of pancreatitis and little evidence of new-onset necrosis. The histological and imaging evidence of fatty replacement and fibrosis progressing to pancreatic atrophy and calcification in CP develops over months to years in spite of ongoing insult from the etiological factors. On the contrary, 15-20% of the AP patients may progress rapidly to a severe form of disease manifesting as organ failure in the setting of pancreatic necrosis [6]. The overall mortality in AP is 2-5% [7] although patients with severe disease may have a mortality up to 30% (5-30%) and the majority of the deaths (50%) occur during the first week [8]. In a meta-analysis of population-based cohort studies, deaths per 100,000 person-years were 1.60 in AP while for CP was 0.09 [9]. Thus, while AP is associated with significantly higher mortality over weeks; the death among CP patients over months to years was infrequent and rarely contributed by acute episode [10]. This discrepancy in the severity of attack between AP and ACP was explained by the presence of increased fibrosis surrounding the intra-pancreatic fat in CP patients. Fibrosis reduces lipolytic flux between acinar cells and adipocytes limiting the acinar adipokines level and also confines the spread of fat necrosis (FN) and peri fat acinar necrosis (PFAN). It contributes to truncated acinar cell necrosis in comparison to AP where the cushion of fibrosis surrounding the pancreas is not available [11].

There is no standard definition of ACP and sparse data exists in the literature regarding the behavior and clinical trend of acute exacerbation of CP as few studies have comprehensively evaluated this entity. Knowledge about the severity and outcome of the disease will help in risk stratification and better management of the patients with ACP. With this background, this study has been undertaken to analyse the epidemiology, clinical profile of the patients, severity of pancreatitis, local/infective/vascular complications and organ failure associated with ACP. Other outcome parameters like hospital stay, intensive care unit (ICU) need, ICU stay, need for percutaneous drain (PCD), endoscopic and surgical intervention, mortality, as well as the trend of the disease on follow-up, were also analysed.

## Materials and methods

The study was conducted in a tertiary care center in north India and consecutive patients of AP with evidence of underlying CP were enrolled prospectively. As there is no standard definition of ACP in the literature, we have used the term ACP to define patients presenting with AP on the basis of revised Atlanta classification [12] requiring hospitalization who have evidence of underlying CP in any of the abdominal imaging including ultrasonography, computed tomography (CT) scan, magnetic cholangio-pancreatography (MRCP) and endoscopic ultrasonography (EUS).

Detailed history and Investigations were carried out to identify the etiology of ACP included liver function test (LFT), fasting triglyceride, serum calcium, parathyroid hormone (PTH), abdominal ultrasonography (USG). All patients without contraindication for CT scan were subjected to contrast-enhanced CT scan of the abdomen and Balthazar score, necrosis score, CT severity index (CTSI) as well as specific features like collections and vascular complications were recorded. When the diagnosis remains elusive after the preliminary investigations patients underwent advanced forms of investigations like MRCP and EUS. Genetic testing for hereditary pancreatitis was not carried out in our study.

Clinical scores like Systemic Inflammatory Response Score (SIRS), Bedside Index for Severity in Acute Pancreatitis (BISAP) were noted at the time of admission [13,14]. Severe pancreatitis was defined by the presence of persistent organ failure (OF), moderately severe pancreatitis as local/systemic complications without persistent OF and mild pancreatitis as the absence of both local and systemic complications [15]. A score of ≥2 in the modified Marshall scoring system for organ dysfunction was defined as the presence of organ failure, and if OF resolved within 48 hours, it was labeled as transient, and when it persisted >48 hours, it was labeled as persistent OF [12,15].

All patients were managed according to standard recommendations, which included fluid resuscitation, organ system support, pain alleviation and nutritional support. Antibiotics were used for suspected pancreatic necrosis infection. Drainage of fluid collections either by percutaneous or endoscopic route was performed in case of persistent OF, suspected infected necrosis, and/or pressure symptoms. Patients failing to recover or worsening with medical management and drainage of collections were subjected to surgical necrosectomy.

Statistical analysis

Statistical analysis was carried out by Microsoft Excel/SPSS software. During analysis of data continuous variables were compared using the student t-test whereas the dichotomous variables were compared using the Chi-square test and descriptive statistics was used as per need. Differences in distribution were tested with the Fisher’s exact test and Chi-square test. The p-value of ≤0.05 was considered statistically significant. Informed consent was obtained from patients before inclusion in our study. The study was undertaken after clearance from the ethical committee of our institute which follows the Helsinki guidelines for research.

## Results

During the study period from January 2015 to June 2016, 45 patients with ACP who fulfilled the inclusion criteria and admitted to our institute were enrolled in the study. The epidemiological and clinical profile of the patients is depicted in Table 1.

**Table 1:** Epidemiological and clinical profile of acute-on-chronic pancreatitis patients

Age (mean ± SD) in years	37.3±13.8 (range 14-72)
Gender (male)	75.6% (n=34)
Etiology	Alcohol 53% (n=24); idiopathic 35% (n=16); gall stone + alcohol4% (n=2); hyperparathyroidism 2% (n=1)
Body mass index	<18 kg/m^2^ - 31% (n=14), 18-24.9 kg/m^2^ - 57.8% (n=26), 25-29.9 kg/m^2^ - 6.7% (n=3), >30 kg/m^2^ ​​​​​​​- 4% (n=2)
Pleural effusion	37.8% (n=17)
Ascites	Mild - 24% (n=11); moderate - 6.7% (n=3); gross - 8.9% (n=4)

Out of 45 patients, four (8.8%) had developed persistent organ failure (OF) while two patients (4.4%) had transient organ failure. Single organ failure was observed in 4.4% (n=2) patients while 2% of patients each had two and three organ failures. Acute interstitial pancreatitis (AIP) was noted in 51% (n=23) patients while 49% (n=22) patients had necrotising pancreatitis involving either pancreatic or extra-pancreatic tissue or both. The majority among the necrotising pancreatitis patients (33%, n=15) had both pancreatic necrosis (PN) as well as extra-pancreatic necrosis (EPN) while 15% (n=7) of the patients had isolated EPN. A local complication of pancreatitis in form of fluid collection on imaging was detected in 82% (n=37) of patients. The majority of the collections (49%, n=22) were seen in association with acute necrotizing pancreatitis (ANP) whereas 33% (n=15) patients had collections in the setting of AIP. A total of 15% (n=7) patients had venous thrombosis out of which splenic vein thrombosis (SVT) was found in 13% (n=6) patients followed by portal vein thrombosis (PVT) in one (2.2%) patient. No patient had pseudoaneurysm on imaging. However, one of the patients had an upper gastrointestinal bleed due to haemosuccus pancreatitis although CT angiography did not reveal any pseudoaneurysm (Table 2).

**Table 2:** Pancreatic characteristics of ACP patients SRIS: Systemic Inflammatory Response Score, BISAP: Bedside Index for Severity in Acute Pancreatitis, OF: organ failure, EPN: extra-pancreatic necrosis, CTSI: CT severity index, CVSF: cardiovascular failure, ALI: acute lung injury, AKI: acute kidney injury, SVT: splenic vein thrombosis, PVT: portal vein thrombosis, APFC: acute peripancreatic fluid collection, WON: walled-off necrosis, ANC: acute necrotic collection.

Severity of pancreatitis	Mild - 17.8% (n=8); moderately severe - 73.3% (n=33); severe - 8.9% (n=4)
Modified Marshall score (mean ± SD)	2.6 ± 1.7
SIRS ≥ 2	6.7% (n=3)
BISAP ≥ 2	22% (n=10)
OF	Persistent OF 8.8% (n=4)
Pulmonary failure	Persistent ALI 6.6% (n=3)
Renal failure	Persistent AKI 4% (n=2)
CVSF	Persistent CVSF 4% (n=2)
Interstitial pancreatitis	51% (n=23)
Necrotising pancreatitis	49% (n=22)
EPN	15.6% (n=7)
Both PN and EPN (EPN+PN)	33% (n=15)
CTSI (mean ± SD)	4.33±2.15 CTSI < 6 – 51% (n=23)
Fluid collection	ANC 22.2% (n=10); APFC 4.4% (n=2); WON 24.4% (n=12); Pseudocyst 26.6% (n=13)
Venous thrombosis	15.5% (n=7) SVT 13% (n=6) PVT 2.2% (n=1)
Arterial pseudo-aneurysm	0
Mortality	4% (n=2)

Four out of five patients who were subjected to PCD had a single PCD and one patient required two PCDs to drain the intra-abdominal collections. PCD insertion was labelled as early when it was inserted within 14 days of pain while insertion of PCD beyond that period was termed as late. Four patients (9%) had early and one patient had late insertion of PCD. PCD insertion successfully resolved organ failure in one patient; however, in another patient, it failed to do so. Surgery in the form of laparotomy and necrosectomy for the management of infected necrosis, compartment syndrome was required in six (13%) patients. One of them required surgery for the associated colonic fistula.

The outcome of the patients was measured by a hospital stay, intensive care unit (ICU) needs, ICU stays, need for ventilatory support, renal replacement therapy (dialysis) and mortality. The mean duration of hospital stay was 8.8±11.9 (range 3-45) days and five of the patients (11%) required ICU admission with a mean ICU stay of 8.2 ±6.9 (range 3-19) days. Ventilatory support was needed in four patients with ALI not improving with oxygen inhalation and the mean ventilator stay was 6.5±3.5 (range 4-9) days. One patient was subjected to renal replacement therapy in the form of peritoneal dialysis. Two patients died secondary to multi-organ dysfunction syndrome (MODS) (Table 3). Thirty-three patients (73%) had no recurrence of pain during the six-months follow-up. Persistent symptoms were noted in 13% (n=6) patients whereas four patients (9%) lost to follow-up.

**Table 3:** Intervention and follow-up of acute-on-chronic pancreatitis patients PCD: percutaneous drain, ERCP: endoscopic retrograde cholangiopancreatography, ICU: intensive care unit.

Number of PCD inserted	11% (n=5)
Indication of PCD insertion	Infected collection 6.6% (n=3); persistent organ failure 4.4% (n=2)
PCD culture	Sterile 8.8% (n=4); *Escherichia coli* 2.2% (n=1)
Antibiotic use	Empirical - 15.6% (n=7); culture guided - 11% (n=5)
ERCP + pancreatic duct stenting	31% (n=14)
Endoscopic cystoduodenostomy	2.2% (n=1)
ERCP + PCD	8.9% (n=4)
Surgery	8.9% (n=4)
ERCP + PCD + surgery	4.4% (n=2)
Hospital stay in days (mean ± SD)	8.8±11.9 (range 3-45)
ICU need	11% (n=5)
Ventilator need	6.6% (n=3)
Ventilator stay in days (mean ± SD)	6.5±3.53 with a range of 4-9 days
Renal replacement therapy (dialysis)	2.2% (n=1) received peritoneal dialysis

## Discussion

In this study, we have evaluated the etiological factors, severity and outcome of patients with ACP who were admitted to our institute. Of the 45 patients analysed, alcohol was the most common etiology (53%) of ACP. The majority of patients in our study had mild or moderately severe pancreatitis and fewer (8.8%) had persistent organ failure. Pancreatic or EPN was observed in around half of the patients. The mean hospital stay was 8.8±11 days with a 4% mortality rate.

The incidence of ACP is rising; probably due to increased use of abdominal imaging in pancreatitis patients as well as technological advances in imaging tools [16,17]. Only one study available in the literature had looked into the clinical profile and outcome of ACP patients [18]. Alcohol was the most common etiology of acute exacerbation and development of CP in our study which was similar to the observations made by Akshintala et al. [18]. This is the probable reason for the male dominance in our study as alcohol intake is less in females in this part of the world.

In a retrospective study done in the western population on ACP, organ failure was noted in 7.2% of patients out of which single organ failure was observed in 6.5% and multi-organ failure was noted in 0.7% of patients [16] which was in comparison to our study having organ failure in 8.8% patients out of which single system involvement in 4.4% and multisystem involvement in 4.4% of patients, indicating that ACP patients have benign disease course [18]. The length of hospital stay, need for intensive unit care and mechanical ventilation were higher in our study compared to previously reported literature in ACP patients, however, the need for renal replacement therapy was similar in both the studies [18]. The mortality rate in our study is higher compare to other studies of ACP (4% vs 0.4%) [18]. This observation indifference is by mere chance as there was a huge difference in the number of patients studied. However, both the studies signify that the patients with ACP had less severe disease and a lower mortality rate compared to patients with AP without CP [18]. The AP episodes with underlying CP have reduced levels of cytokines like tumor necrosis factor α leading into less systemic inflammation and organ failure in comparison to AP without underlying CP [19]. This discrepancy in the severity of attack between ACP and AP without CP can be also explained by the presence of increased fibrosis surrounding the intra-pancreatic fat in CP patients reducing lipolytic flux between acinar cells and adipocytes and also confines the spread of FN and PFAN [11].

Our study is the first study to report all local complications of ACP patients. Pancreatic or EPN was observed in around 50% of patients which was lower than the reported literature of AP without CP (77.5%) from the same institute [20]. Local complications in the form of fluid collections were observed in association with necrotising pancreatitis in most of our patients. Drainage of pancreatic fluid collection (percutaneous catheter drainage as well as transmural drainage) was required as part of management in 13% of patients which was significantly less compared to AP patients without CP (55-87%), however, the need for surgical necrosectomy was similar between the study groups [20,21].

Our study had certain limitations, we did not perform a genetic study for evaluation of the cause of pancreatitis. This study is from a single center and the number of patients included was comparatively less. However, this study will stimulate researchers to conduct a multicenter study with a large number of patients to ascertain the epidemiology, clinical profile and outcome of ACP patients.

## Conclusions

In summary, ACP patients experience a milder disease course and less likely to die due to an AP attack. Hence, it is necessary to identify ACP as a different clinical entity separate from AP without CP. Knowledge about the epidemiology of disease will help in the early elimination of the causative factors and further improve the outcome of the patients with ACP.
